# Bioprocessing of tea oil fruit hull with acetic acid organosolv pretreatment in combination with alkaline H_2_O_2_

**DOI:** 10.1186/s13068-017-0777-1

**Published:** 2017-04-08

**Authors:** Song Tang, Rukuan Liu, Fubao Fuelbiol Sun, Chunying Dong, Rui Wang, Zhongyuan Gao, Zhanying Zhang, Zhihong Xiao, Changzhu Li, Hui Li

**Affiliations:** 1 Key Laboratory of Carbohydrate Chemistry and Biotechnology, Ministry of Education, School of Biotechnology, Jiangnan University, Wuxi, 214122 China; 2 State Key Laboratory of Pulp and Paper Engineering, South China University of Technology, Guangzhou, 510640 China; 3 National Engineering Research Center for Oil-tea Camellia, Hunan Academy of Forestry, Changsha, 410004 China; 4 Key Laboratory of Advanced Textile Materials and Manufacturing Technology, Ministry of Education, Zhejiang Sci-Tech University, Hangzhou, 310018 China; 5 Centre for Tropical Crops and Biocommodities, Queensland University of Technology, Brisbane, QLD 4001 Australia

**Keywords:** Tea oil fruit hull, Acetic acid, Alkaline H_2_O_2_, Mild pretreatment, Delignification, Enzymatic hydrolysis

## Abstract

**Background:**

As a natural renewable biomass, the tea oil fruit hull (TOFH) mainly consists of lignocellulose, together with some bioactive substances. Our earlier work constructed a two-stage solvent-based process, including one aqueous ethanol organosolv extraction and an atmospheric glycerol organosolv (AGO) pretreatment, for bioprocessing of the TOFH into diverse bioproducts. However, the AGO pretreatment is not as selective as expected in removing the lignin from TOFH, resulting in the limited delignification and simultaneously high cellulose loss.

**Results:**

In this study, acetic acid organosolv (AAO) pretreatment was optimized with experimental design to fractionate the TOFH selectively. Alkaline hydrogen peroxide (AHP) pretreatment was used for further delignification. Results indicate that the AAO–AHP pretreatment had an extremely good selectivity at component fractionation, resulting in 92% delignification and 88% hemicellulose removal, with 87% cellulose retention. The pretreated substrate presented a remarkable enzymatic hydrolysis of 85% for 48 h at a low cellulase loading of 3 FPU/g dry mass. The hydrolyzability was correlated with the composition and structure of substrates by using scanning electron microscopy, confocal laser scanning microscopy, and X-ray diffraction.

**Conclusion:**

The mild AAO–AHP pretreatment is an environmentally benign and advantageous scheme for biorefinery of the agroforestry biomass into value-added bioproducts.

**Electronic supplementary material:**

The online version of this article (doi:10.1186/s13068-017-0777-1) contains supplementary material, which is available to authorized users.

## Background

*Camellia oleifera* Abel., originated from China at least as early as 2300 years ago, is an evergreen boscage or small tree in Camellia family. Currently, it is widely planted in some Asian countries, predominantly in China. As an edible oil crop, its seed is mainly used for extruding nourishing oils enriched with unsaturated fatty acids, high up to 90%, which is reported to be the highest content in edible oils [1]. The unsaturated fatty acids mainly consist of oleic acid and linoleic acid, in which the oleic acid content (75‒87%) is 5‒10% higher than that of the olive oil [2]. Thus, the cooking oil, known as “eastern olive oil” in China, almost has met the international nutritional standards of “omega meals.” Accordingly, the *Camellia oleifera* Abel. crop has attracted huge economic interests in China. In recent years, the plantation of *C. oleifera* has increased by a yearly rate of 5.4% [3]. As byproducts from the tea oil processing industry, the outputs of tea oil fruit hull (TOFH), seed shell, and oil cake have reached around 5 million tons every year [4]. It has been estimated that more than 10 million tons of tea oil processing byproducts will be generated with a plantation area of 6 million hectares by 2020 [1].

Traditionally, these byproducts are discarded away or burned up in the tea oil processing industry, which not only causes environmental concerns but also is a waste of bioresources. Considering of rich bioactive substances in the seed shell and oil cake, much attention has been paid to valorization of these byproducts [3, 5]. However, the use of TOFH is very limited, though it accounts for ~75% of these total byproducts [6]. As a natural lignocellulosic biomass, the TOFH consists of 12‒14% cellulose, 19‒21% hemicellulose, and 26‒27% lignin, in addition to the rich bioactive substances (i.e., tea saponin and tannin) [5, 7]. Attempts have been made to process *C. oleifera* Abel hull into diverse bioproducts using a two-stage solvent-based process, which includes one aqueous ethanol organosolv (AEO) extraction step, followed by an atmospheric glycerol organosolv (AGO) pretreatment [7]. With the mild AEO treatment, the extraction of tea saponin and tannin reached above 80%, respectively. However, the severe AGO pretreatment (180 °C, 3 h) is not so selective as expected in altering the main composition, resulting in a low delignification (34%), a low hemicellulose removal (40%), but a high cellulose loss (18%) from the TOFH. Thus, more efforts are addressed to find an alternative method to pretreat the AEO-extracted TOFH.

As a common organic solvent, acetic acid organosolv (AAO) pretreatment has been proved to be efficient in selectively fractionating the lignocellulosic biomass into cellulose pulp, lignin, and hemicellulose under mild conditions [8–11]. The AAO lignin is desirable for many important applications due to its low molecular weight and high reactivity [8, 12]. Pan and Sano [9] removed 75% of lignin and 83% of hemicellulose from wheat straw under atmospheric AAO pretreatment condition (105 °C, 3 h, 4% H_2_SO_4_). Atmospheric AAO pretreatment has some appealing advantages, i.e., mild process and good selectivity. Although AAO pretreatment has been applied on common lignocellulosic biomass (rice straw, wheat straw, sugarcane bagasse, and hard wood), it has not been evaluated yet on TOFH.

On the other hand, alkaline hydrogen peroxide (AHP) pretreatment can lead to significantly improved hydrolyzability by further delignification [13, 14]. The AHP pretreatment has gained an increasing interest in the further delignification by combination with other pretreatment methods [11], i.e., steam explosion [15], dilute acid [16–18], concentrated phosphoric acid [19], and alkaline [20, 21]. Zhu et al. [15] removed 92% lignin from *E. ulmoides* wood with the steam explosion pretreatment followed by AHP, and thereby achieved a high glucose yield of above 90%. To our knowledge, there is very little information reported to date on a combination of the AAO pretreatment with the AHP acting on the lignocellulosic biomass [18, 22].

In this study, an AAO pretreatment followed by AHP was evaluated as a mild process to fractionate the AEO-extracted TOFH selectively. Firstly, the mild AAO process was optimized with a statistical analysis to find key variables determinant to the good selectivity and hydrolyzability. Secondly, the AHP pretreatment was used to further improve the enzymatic hydrolysis of AAO-pretreated substrates. Structural features were characterized with modern analytic equipment including scanning electron microscopy (SEM), confocal laser scanning microscopy (CLSM), and X-ray diffraction (XRD). The fractionation of AAO- and AAO–AHP-pretreated substrates was correlated with these physio-chemical features. Finally, mass balance analysis was conducted for the mild AAO–AHP pretreatment process.

## Results and discussion

### Construction of the AAO pretreatment process

Numerous researchers have demonstrated that variables such as pretreatment temperature, pretreatment time, acetic acid concentration, and acid catalyst contribute to the AAO pretreatment of lignocellulosic biomass [8, 23]. Accordingly, these variables were taken into careful consideration by using Plackett–Burman design (PBD), steepest ascent design, and central composite design (CCD) in this study.

According to the PBD (Table 1; Additional file 1: Tables S1 and S2), these variables had a significant effect (*P* < 0.05) on the pretreatment except cellulose retention. Given that lignin impedes substantially substrate hydrolyzability through non-productive binding of cellulase enzymes to its surface and/or through steric hindrance [24], the delignification was taken as an exclusive dependent variable for AAO pretreatment. In addition to the pretreatment temperature, the other three variables were taken into consideration to maximize a delignification from the lignocellulosic biomass. Based on the steepest ascent design experiment (Table 2), the pretreatment condition (acetic acid 50%, pretreatment time 1.63 h and H_2_SO_4_ addition 0.58%) was selected as the central point of the CCD for further optimization using CCD (Additional file 1: Tables S3–S6).

**Table 1:** PBD experimental result of the AAO pretreatment

Runs	*X*_1_	*X*_2_	*X*_3_	*X*_4_	Response (%)			
					Cellulose retention	Hemicellulose retention	Delignification	Pretreatment yield
1	0.5	20	0.7	135	90.2	91.0	11.9	85.9
2	2.0	20	0.2	115	91.3	95.7	7.3	87.8
3	0.5	60	0.7	135	96.8	19.8	67.1	35.9
4	0.5	60	0.2	115	88.3	94.8	9.6	94.7
5	2.0	20	0.7	135	90.8	11.4	33.8	39.9
6	0.5	20	0.7	115	89.0	93.0	11.4	88.0
7	2.0	60	0.2	135	92.2	28.7	52.9	42.7
8	2.0	60	0.7	115	96.3	21.6	60.0	33.4
9	2.0	60	0.7	115	96.0	22.8	58.9	40.7
10	0.5	20	0.2	115	90.0	95.8	9.5	94.1
11	0.5	60	0.2	135	87.7	87.7	23.1	80.6
12	2.0	20	0.2	135	91.3	48.3	27.0	57.7

*X*_*1*_ time, *X*_*2*_ acetic acid concentrations, *X*_*3*_ H_2_SO_4_ addition, *X*_*4*_ pretreatment temperature

**Table 2:** Steepest ascent experiment of AAO pretreatment

Runs	*X*_1_	*X*_2_	*X*_3_	Delignification (%)
	Time (h)	Acetic acid (%)	H_2_SO_4_ addition (%)	
1	1.3	40	0.45	23.2
2	1.4	45	0.51	37.7
3	1.6	50	0.58	60.1
4	1.8	55	0.64	64.1
5	2.0	60	0.70	66.3
6	2.2	65	0.76	67.3
7	2.4	70	0.83	68.3

For the CCD, 20 experiments were carried out (Additional file 1: Table S4). The regression model was highly statistically significant (*P* < 0.0001), but the lack of fit was not significant (*P* = 0.357 > 0.1) (Additional file 1: Table S5). Meanwhile, relatively lower value (1.97 < 10%) of variation coefficient indicated the good precision and reliability of these experiments. Adequate precision for our model had a signal-to-noise ratio of 34.2 (>4), meaning an adequate signal. Under these circumstances, each variable and the interaction of every two variables also had a remarkable effect (*P* < 0.05) (Additional file 1: Table S6). The best model was identified using the coefficient of determination *R* ^2^ (0.989), suggesting that 98.9% of the sample variation was attributed to the variables. For a regression model, the present *R* ^2^ value (0.978) reflected a very good fit of the observed and predicted responses, implying that the model is reliable for delignification with AAO pretreatment (Additional file 1: Figure S1). The maximum delignification obtained by using above selected variables was 70.0%, and the experimental maximum obtained was 69.0 ± 0.6%. The data show that predicted data on the response from empirical model were in agreement with those observed in the range of the operating variables. The coefficients of regression were calculated, and the following regression equation was obtained.

1 $$ Y = { 4}. 9 1 { } + { 4}. 8 8 { } \times X_{1} + { 3}. 7 4 { } \times X_{2} + { 5}. 7 2 { } \times X_{3} + \, 0. 4 2 { } \times X_{1} \times X_{2} {-}{ 1}. 7 4 { } \times X_{1} \times X_{3} {-}{ 1}. 6 2 { } \times X_{2} \times X_{3} {-}{ 2}. 7 9 { } \times X_{1}^{2} {-}{ 2}. 3 4 { } \times X_{2}^{2} {-}{ 1}. 9 8 { } \times X_{3}^{2} $$

where *Y* = Response (Delignification), *X*_1_ = Pretreatment time, *X*_2_ = Acetic acid concentration, and *X*_3_ = H_2_SO_4_ addition in coded values.

**Fig. 1:**
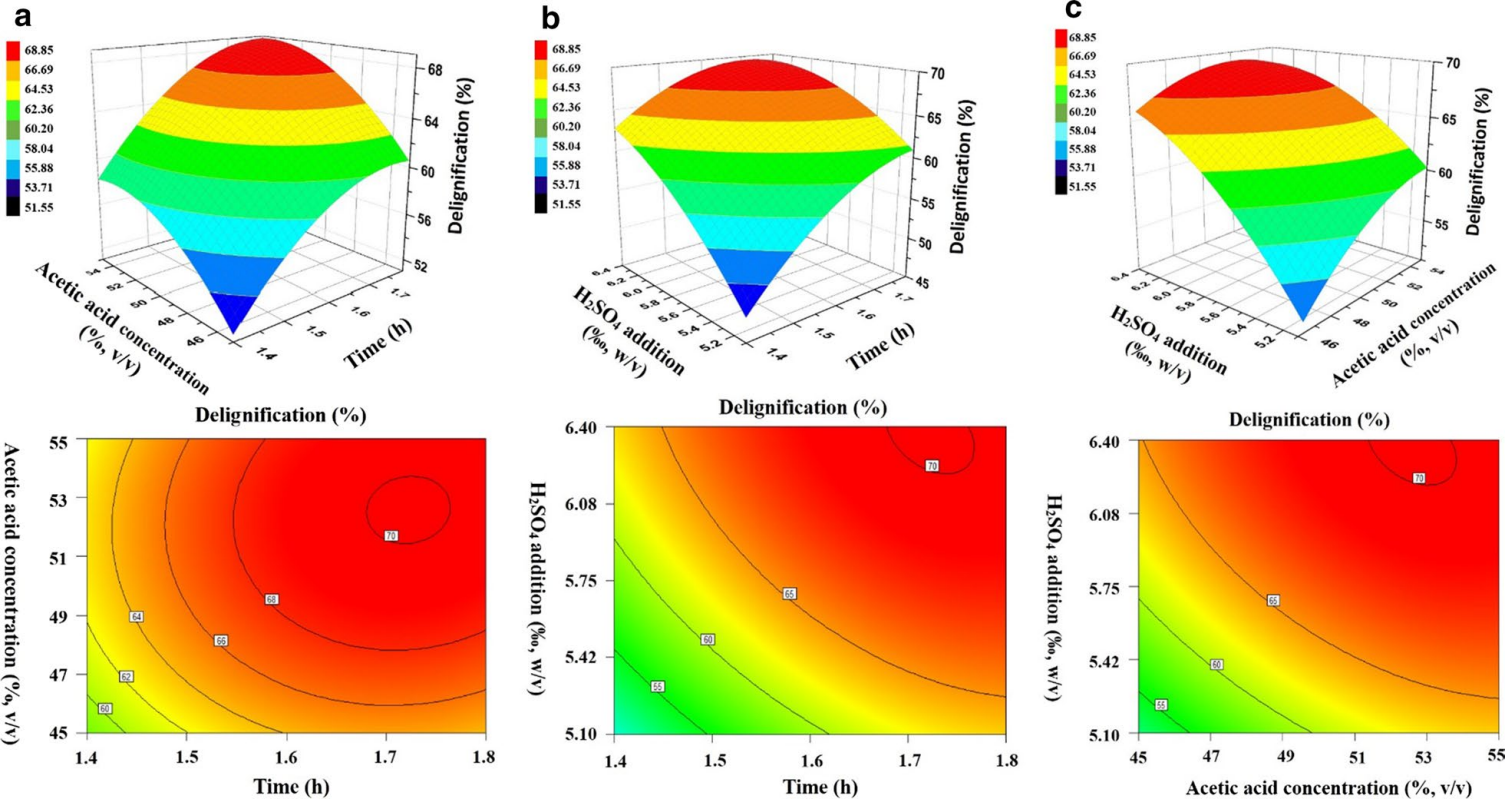
Response surface plot (3D and 2D) for the interactive effect of variables. **a** Effect of acetic acid concentration and pretreatment time, fixed H_2_SO_4_ addition at 0.64 (%, w/v); **b** effect of H_2_SO_4_ addition and pretreatment time, fixed acetic acid concentration at 52.6 (%, v/v); **c** effect of H_2_SO_4_ addition and acetic acid concentration, fixed pretreatment time at 1.7 h

To understand that the effect of these variables on the delignification during the AAO pretreatment, the predicted model was presented as 3D/2D response surface graphs, as shown in Fig. 1. These variables were optimized as follows: pretreatment time 1.72 h, H_2_SO_4_ addition 0.64%, and acetic acid content 52.6% for the AAO pretreatment. Under optimized condition, the removal of lignin and hemicellulose reached 68 and 86%, respectively. It means that there were 44.3% cellulose, 11.8% hemicellulose, and 25.7% lignin existing in the AAO-pretreated substrate. The result is comparable to that reported elsewhere on the AAO pretreatment (Table 3). Like other common (agricultural or woody) lignocellulosic biomass, the AAO pretreatment has selectively removed most of the lignin and hemicellulose from the TOFH with an almost intact cellulose retention [12]. Notably, the optimal pretreatment conditions mentioned in these publications are obviously different, which is seemingly due to the variety of lignocellulosic feedstock. These results have indicated that the AAO pretreatment is as effective for selectively disintegrating the lignocellulosic byproduct of tea oil processing industry as other lignocellulosic biomass.

**Table 3:** Comparison of the AAO–AHP pretreatment with others on lignocellulosic biomass

Feedstock	Pretreatment process						Resulting solids			Source
	Type	Pretreatment				Post-pretreatment	C	H	L	
		Content (%)	Catalyst	T (°C)	Time (h)		%			
*C. inophyllum* shell	H_2_SO_4_ + NaOH	1%	At 90 °C for a fixed time				35	7	25	Cheng et al. [26]
*E. ulmoides* Oliver	SEP + AHP	–	–	213	1/3	1.5% H_2_O_2_, pH 11.5	77	4	5	Zhu et al. [15]
*Lespedeza* stalk	SEP + AHP	–	–	184	1/15	2% H_2_O_2_, pH 11.5	83	0	10	Su et al. [27]
EFB	AAO + AQA	–	7 (15) % AAO (AQA) pretreatment at 180 °C for 15 min				65	22	21	Kim et al. [22]
Wheat straw	AAO	90	0.4% H_2_SO_4_	105	3	–	67	11	4	Pan and Sano [9]
Wheat straw	AAO	80	8.5% HNO_3_	120	0.3	–	96	3	1	Sun et al. [38]
Sugarcane straw	AAO	80	0.3% HCl	120	3	–	61	7	12	Saad et al. [23]
Sugarcane bagasse	AAO	90	0.1% H_2_SO_4_	105	3	–	64	17	13	Zhao et al. [10]
Beech	AAO	90	0.2% HCl	130	1	–	77	8	8	Vila et al. [43]
Corn stover	HCl + H_2_O_2_	–	7% HCl	120	2/3	3% H_2_O_2_ + 0.1% FeSO_2_	26	2	12	Li et al. [16]
Wheat straw	AAO + AHP	55%	30% FA	105	3	1% H_2_O_2_	70	15	2	Snelders et al. [18]
TOFH	AGO	70	–	180	3		22	26	27	Sun et al. [7]
TOFH	AAO	53	0.6% H_2_SO_4_	125	1.7	–	44	12	26	This study
TOFH	AAO + AHP	53	0.6% H_2_SO_4_	125	1.7	3% H_2_O_2_, pH 11.5	65	15	10	This study

*EFB* empty fruit bunches, *SEP* steam explosion pretreatment, *AQA* aqueous ammonia, *FA* formic acid, *C* cellulose content, *H* hemicellulose content, *L* lignin content

### Enzymatic hydrolysis of AAO-pretreated substrates

**Fig. 2:**
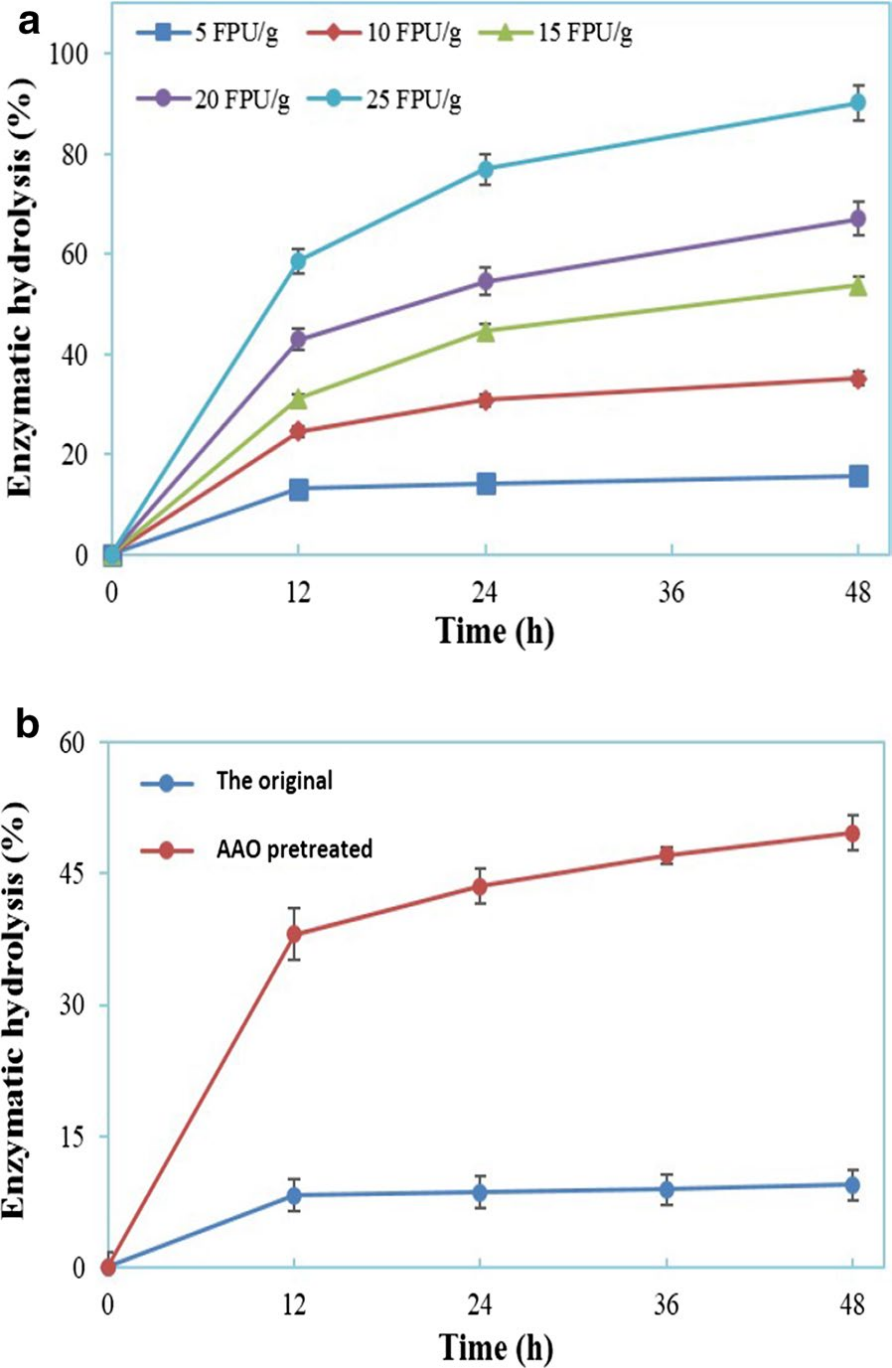
Evaluation of the hydrolyzability of AAO-pretreated substrates. **a** Effect of different enzyme loadings (FPU/g DM) on the enzyme hydrolysis of substrates (2% solid content); **b** enzyme hydrolysis (5% solid content, 15 FPU/g DM) of substrates before and after the AAO pretreatment

Enzymatic hydrolysis of the AAO-pretreated substrates was used to evaluate for its hydrolyzability in this experiment. As shown in Fig. 2a, the enzymatic hydrolysis of AAO-pretreated substrates increased with a big enzyme loading, reaching ~90% for 48 h at 25 FPU/g DM. The result indicated that the lignocellulosic byproduct of tea oil processing industry was applicable as the feedstock for bioconversion, like other common lignocellulosic biomass [9, 22]. As compared to that before the AAO pretreatment (Fig. 2b), the enzymatic hydrolysis of AAO-pretreated substrates enhanced by more than three times, indicating a remarkable improvement of the substrate hydrolyzability.

Nevertheless, the hydrolyzability of AAO-pretreated TOFH was still limited, far away from the industrial interest, as the high enzymatic hydrolysis was with sacrifice of high enzyme loading. As for the AAO-pretreated substrate, it still had such a high lignin content up to 25% that the lignin could impede the enzymatic hydrolyzability of substrates to some extent. Additionally, acetylization of the cellulose, occurring commonly during the AAO pretreatment, was very probably adverse to the enzymatic hydrolysis of substrates [10, 25]. Therefore, a second-step AHP pretreatment was implemented to improve the enzymatic hydrolysis with a low cellulase loading.

### AHP pretreatment for the further delignification

**Fig. 3:**
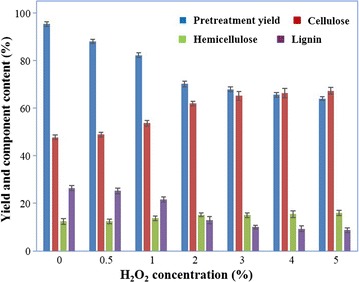
Selection of H_2_O_2_ concentration (v/v on the solution) for the AHP pretreatment. The pretreatment was carried out for 12 h at a fixed condition (pH 11.5, 25 °C, 12.5% solid content, 150 rpm shaking speed). The percentages of yield and component content refer to the weights of AAO-pretreated substrates and AHP-pretreated substrates, respectively

During the AHP pretreatment process, H_2_O_2_ concentration was selected as the key variable under some fixed conditions (12.5% solid content, pH 11.5 and ambient temperature), since delignification is strongly pH-dependent. AHP pretreatment was evaluated at different H_2_O_2_ concentrations, as shown in Fig. 3. Initially, the pretreatment yield reduced obviously at a high H_2_O_2_ concentration, owing to delignification. As a result, cellulose content of AHP-pretreated substrate increased significantly. At 3% of H_2_O_2_, the substrate had a high cellulose content with almost a minimum lignin residual, accounting for 65 and 10% of the substrate composition, respectively. Thereafter, the lignin removal increased marginally and thus contributed to a slight increase of cellulose content. Consequently, 3% H_2_O_2_ was selected for the optimal addition for the AHP pretreatment. Meanwhile, the cellulose retention and the delignification reached 98 and 74%, respectively. And the AAO–AHP-pretreated substrate is cellulose-enriched, consisting of 65% cellulose, 15% hemicellulose, and 10% lignin. The data indicated that the AHP pretreatment, commonly used as a post-pretreatment, had an outstanding capability of selective delignification. This is in agreement with the literature [14–16, 18].

As for the AAO–AHP pretreatment, the lignin and hemicellulose removal was 92 and 88%, respectively, with 87% of the cellulose retention. The result is extremely competitive to other studies published on the uncommon feedstock and mild AAO-/AHP-based pretreatment. As shown in Table 3, some lignocellulosic feedstocks, i.e., *C. inophyllum* shell and *E. ulmoides* Oliver have gained much research interest [15, 26]. Like these uncommon lignocellulosic materials, TOFH is applicable as a value-added lignocellulosic feedstock for the future biofuel production. In most case, the single AAO pretreatment with acid catalysis is not satisfactory as expected because of the limited delignification and cellulose acetylization [9, 10, 23]. In our study, the AAO pretreatment removed 70% lignin from TOFH, contributing the residual lignin to 26% of the pretreated substrate. Thus, a subsequent post-pretreatment is desirable for further delignification to release more fermentable sugars. AHP has been confirmed to be a very effective post-pretreatment by dozens of researchers [15, 27]. Zhu et al. [15] studied the AHP pretreatment (1.5% H_2_O_2_, pH 11.5) of steam exploded *E. ulmoides* Oliver and achieved a good delignification of 92%. An AAO–NH_3_·H_2_O combination process developed by Kim et al. [22] supplied a guideline to find new pretreatment, though presenting a limited component selectivity (60% delignification and 67% cellulose retention) on empty fruit bunches probably due to the low solvent concentration. At contrast, Snelders et al. [18] contributed to 96% delignification and 93% cellulose retention in the wheat straw by using a formic/acetic acid–H_2_O_2_ pretreatment, indicating of a robust pretreatment selectivity. The AAO–AHP pretreatment developed herein removed 92% lignin and 88% hemicellulose from the TOFH, simultaneously with 87% cellulose retention. The pretreatment produced a cellulose-rich (65%) substrate with a low lignin content (10%). It was confirmed that the AAO–AHP pretreatment was effective in selectively pretreating the lignocellulosic biomass. In our recent publication on AGO pretreatment of TOFH, the AGO-pretreated substrate had a low cellulose content of 22% and high lignin content of 27% [7]. Comparing the AGO pretreatment, the AAO–AHP pretreatment developed herein has presented a good advantage over it.

### Enzymatic hydrolysis of substrates after AAO–AHP pretreatment

**Fig. 4:**
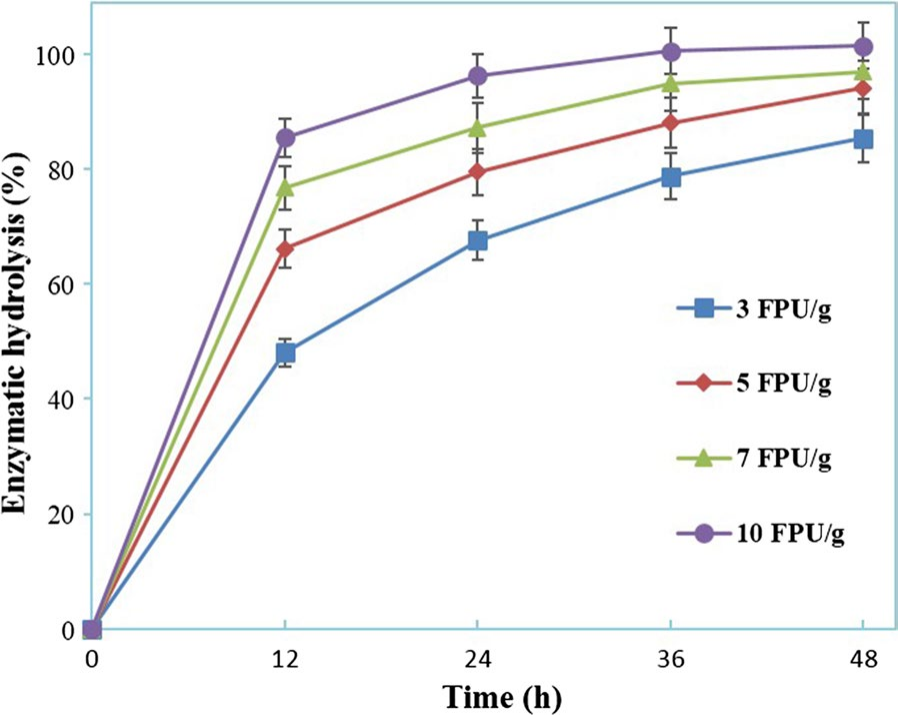
Enzymatic hydrolysis of AAO–AHP-pretreated substrates at different enzyme loadings (FPU/g DM). The hydrolysis was carried out at pH 4.8, 50 °C for 48 h with 5% solid content (w/w)

Enzymatic hydrolysis of AAO–AHP-pretreated substrates was implemented at 5% solid content for 48 h to evaluate the hydrolyzability. As shown in Fig. 4, the substrate achieved an almost complete hydrolysis at 10 FPU/g DM. The 48-h enzymatic hydrolysis of substrates was 85 and 94% at 3 and 5 FPU/g, respectively. This was much higher as compared to that of the AAO-pretreated substrates, in that the latter was only 20% of the enzymatic hydrolysis at 5 FPU/g DM with 2% solid content. Based on the above chemical composition, it can be judged that the increase of enzymatic hydrolysis originated from the further delignification made by AHP pretreatment. Additionally, the AHP pretreatment can effectively remove acetyl groups formed with acetic acid acetylizing the cellulose during the above AAO pretreatment, which is at least partly responsible for the improved hydrolyzability [10].

The hydrolyzability of AAO–AHP-pretreated substrates was compared with that reported on other uncommon lignocellulosic biomass and on other AAO-/AHP-based pretreatment (Table 4). Like other lignocellulosic substrate, the hydrolyzability of the original TOFH is very poor, hence a low enzymatic hydrolysis even with a high cellulase loading, because of the recalcitrant structure [26, 28, 29]. The substrates undergoing AAO-/AHP-based pretreatment have presented an improved hydrolyzability to a different extent depending on the substrate variety, pretreatment process, hydrolytic enzymes, and hydrolytic conditions [14, 15, 22, 26]. It is evident that the AAO–AHP-pretreated TOFH substrate is competitive to these substrates at the hydrolyzability.

**Table 4:** Hydrolyzability of various substrates

Substrate		Hydrolytic condition				Enzymatic hydrolysis (%)	Source
Variety	Pretreatment type	Solid	Cellulase		Time		
		%	Variety (FPU/g)	Loading	h		
*C. inophyllum* shell	H_2_SO_4_ + NaOH	10	Accellerase™ 1500	–	72	50 (RS)	Cheng et al. [26]
Bamboo shoot hull	9% Na_3_PO_4_ +3% H_2_O_2_	1	Accellerase™ 1500	30	72	86 (RS)	Qing et al. [14]
EFB	AAO − AQA	2	Cellulase 1.5 L	15	96	73	Kim et al. [22]
*E. ulmoides* Oliver	SEP + AHP	5	Youtell cellulase	20	96	83 (G)	Zhu et al. [15]
Cashew apple bagasse	4.3% AHP	9	Novozymes cellulase	15	72	86 (G)	da Costa et al. [13]
*Lespedeza* stalk	SEP + AHP	5	Cellulase 1.5 L	12.4	96	89 (G)	Su et al. [27]
Sugarcane bagasse	AAO	2.5	Cellulase 1.5 L	20	120	63 (RS)	Zhao et al. [10]
Corn stover	3% H_2_O_2_ + 7.5 g/L NaOH	6	Spezyme CP	30	72	35(G)	He et al. [21]
Corn stover	HCl + H_2_O_2_	5	Novozymes cellulase	3	72	71 (G)	Li et al. [16]
Sugarcane bagasse	2% H_2_SO_4_ + 4.7% H_2_O_2_	10	Celluclast 1.5 L	4.1	120	65 (G)	Morando et al. [17]
TOFH	None	5	GC220	50	48	39 (RS)	Sun et al. [7]
TOFH	AGO	5	GC220	20	48	80 (RS)	Sun et al. [7]
TOFH	AAO	5	Cellic CTec2	15	48	50 (RS)	This paper
TOFH	AAO + AHP	5	Cellic CTec2	3	48	85 (RS)	This paper

*EFB* empty fruit bunches, *SEP* steam explosion pretreatment, *AQA* aqueous ammonia, *G* glucose, *RS* reducing sugars

As for the pretreatment, all these methods can allow the substrate for hydrolyzability increase with varying degrees [10, 21, 27, 30]. Notably, Li et al. [16] reported 71% of enzymatic hydrolysis at an industrially relevant enzyme loading (3 FPU/g substrate) when evaluating the hydrolyzability of dilute HCl–H_2_O_2_-pretreated corn stover. In this study, the enzymatic hydrolysis of TOFH subjected to AAO–AHP pretreatment reached 85% at 3 FPU/g of cellulase preparation Cellic CTec2. Such low enzyme loading has been considered to be very economic at present enzyme costs [16]. Compared with the AGO-pretreated TOFH reported by us just recently [7], the enzyme loading on AAO–AHP-pretreated substrates was only 15% that of it (20 FPU/g) to reach almost equivalent enzymatic hydrolysis. Accordingly, it is certain that the hydrolyzability of AAO–AHP-pretreated TOFH feedstock is robust. TOFH, as a main byproduct of tea oil processing industry, is applicable for the current biofuel production. The AAO–AHP combination pretreatment can be a promising candidate for bioprocessing of lignocellulosic biomass.

### Structural features of AAO and AAO–AHP-pretreated substrates

Many researchers have argued that the main chemical composition (cellulose, lignin, and hemicellulose) and physical structure (surface area, average size, and the crystallinity) of lignocellulose biomass are available to represent the inherent recalcitrance of substrate to enzymatic hydrolysis [29, 31–33]. Consequently, structural features of substrates at different pretreatment stages were depicted in the ensuing work.

### SEM analysis

As for exploring the physical feature of AAO and AAO–AHP-pretreated substrates in favor of enzymatic hydrolyzability, some morphological changes of feedstock with different pretreatments were determined with a series of comparative SEM observations (Additional file 1: Figure S2). The AEO undissolved feedstock exposed the inner structure of lignocellulose presenting more bunchy fiber and curly surface clumps. With the two-stage pretreatment, a significant size reduction of lignocellulose occurred. On the surface of AAO-pretreated substrates, there existed so many holes, very probably due to the AAO penetration and solvolytic reaction during the pretreatment, which increased the specific surface area of substrates and facilitated the accessibility to cellulase enzymes. After the AHP pretreatment, the perforated surface was further disrupted in some small fragments with a more roughness and surface area. These images indicate that the pretreatment has gradually dissected the physically structural barrier of substrates and led to a large portion of long defibrillated fibrils, making the cellulose more exposed with more surface area and roughness. Consequently, the surface feature of two-stage pretreated substrates is extremely accessible and susceptible to cellulase enzymes, hence to a high enzymatic hydrolysis yield [14, 33–35]. Therefore, these observations partly explain why the pretreatment can effectively improve the enzymatic hydrolyzability of the TOFH.

### CLSM analysis

**Fig. 5:**
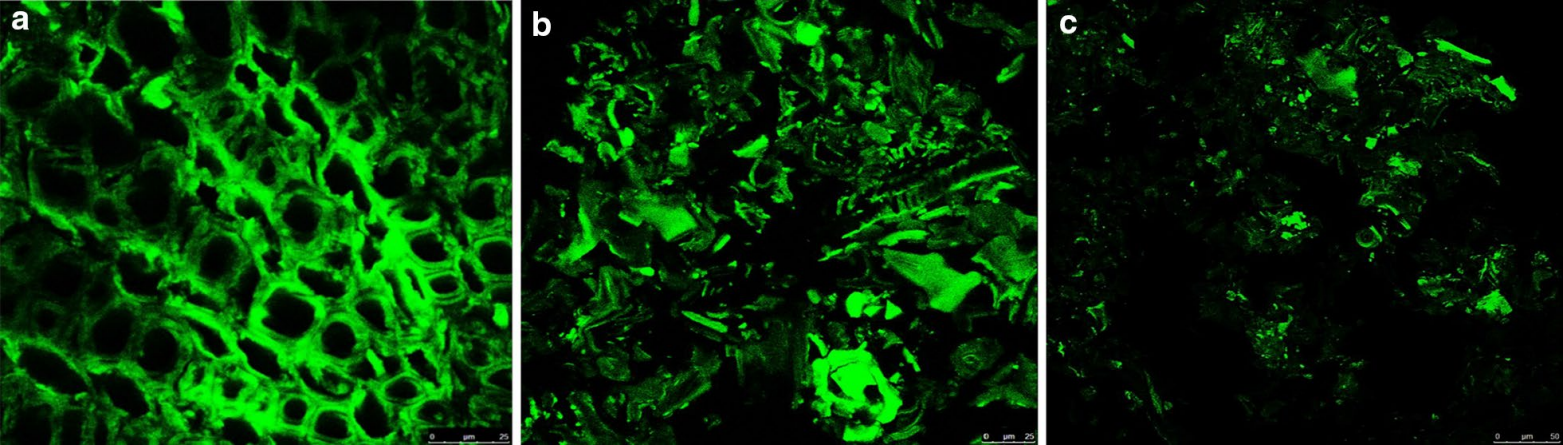
CLSM images of material before and after the AAO and AAO–AHP pretreatment. *Scale bar* 25 μm. **a**–**c** means the original, AAO-pretreated and AAO–AHP-pretreated substance, respectively

Considering the lignin is a complex heteropolymer with strong autofluorescence in the visible and far-IR regions, the CLSM was used to visualize the structure on the lignin distribution, cell wall transverse dimensions, and fiber surfaces [32, 33]. Figure 5 illustrates the CLSM images of substance undergoing the AAO pretreatment in combination with alkaline H_2_O_2_. The AEO undissolved feedstock had an intact structure with the lignin-rich sclerenchyma and middle lamella. After the AAO pretreatment, the initial intact structure was disrupted. The primary and secondary cell walls were separated from the middle lamella, resulting in a clear distortion of the whole lignocellulosic structure at a tissue and organ level. These visualized phenomena are highlighted with the subsequent AHP pretreatment. With the AAO–AHP pretreatment, the sclerenchyma and middle lamella disintegrated, and the distorted structure was broken into more fine fragments with a more roughness and surface area. The observation is in accordance with the above SEM images.

Additionally, the brightness of fluorescence that means lignin distribution became weak with the pretreatment process. After the AAO–AHP pretreatment, many ultrastructures of substrates were invisible with too low lignin distribution. The observation was consistent with the above analysis of component contents.

### XRD analysis

As shown in Additional file 1: Figure S3, X-ray diffraction curves of the feedstock before and after two-stage pretreatment were used to monitor the crystallinity. The XRD pattern had three typical peaks of cellulose, at 16° (10ī), 21.9° (002), and 34.6° (040). [36]. With the AAO–AHP pretreatment, the position (002) of crystal face diffraction peak shifted to the right obviously, while the other two (10ī, 040) kept stable. And the peaks of the crystal surface at the position (10ī, 002) intensified significantly. The result inferred these pretreatments had a remarkable influence on the crystalline cellulose, at least causing an increase in crystalline cellulose content. To verify it, the crystallinity index (CrI) and crystallite size of samples were detected (Additional file 1: Table S7). The CrI was 21.3% in the original feedstock. It augmented to 41.3 and 52.1%, respectively, with the AAO and AAO–AHP pretreatment. This relative increase indicates that the removal of amorphous components such as lignin and hemicelluloses (resulting in an increase of CrI) outplayed the swelling and dissolution of the cellulose (a reduction of CrI) [31, 33]. As for the crystallite size, the average size of the original cellulose crystallite was 1.5 nm (002). Notably, it became big slightly after the AAO pretreatment. And the average size increased twice with the AHP pretreatment. It is apparent that the pretreatment resulted in a big size of crystalline cellulose. The result is beyond the expectation and inconsistent with previous results that the pretreatment can dissociate the crystalline cellulose into some small sizes [33, 37]. This finding is so abnormal that scare information is responsible for it [36]. Based on the significant peak (002) shift from 21.1° to 22.5°, it can be guessed that re-formation or recrystallization of crystalline cellulose possibly occurred during the AHP pretreatment [31–33].

Based on the above structural observation, it is revealed that the AAO–AHP pretreatment can contribute to the great modification of lignocellulosic biomass at surface area, average size, components redistribution to a good enzymatic hydrolysis.

### Mass balance analysis

**Fig. 6:**
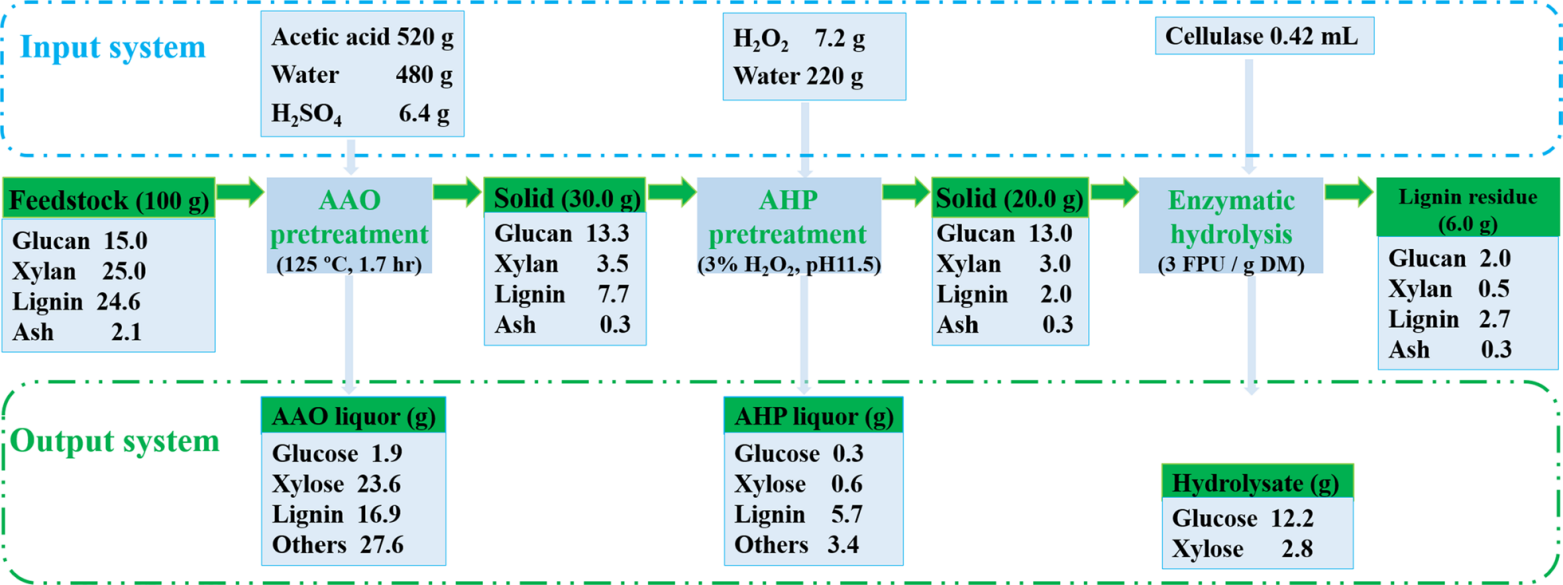
Mass balance of the AAO–AHP pretreatment of TOFH

The analysis presents an entire workflow on the two-stage fractionation of TOFH. As shown in Fig. 6, 70% of the lignin and 86% of the hemicellulose removed from the original feedstock with AAO pretreatment. The AAO-pretreated substrate mainly consisted of 44% cellulose, 12% hemicellulose, and 25% lignin. Furthermore, the AHP pretreatment enabled the lignocellulosic substrate to a deep delignification selectively, contributing to 75% of the lignin removal with an almost intact cellulose retainment. In other words, the AAO–AHP pretreatment has extracted 92% of the lignin and 88% of the hemicelluloses from the original feedstock, with 87% the cellulose retention. As a result, the feedstock undergoing the two-stage pretreatment is typical of rich cellulose content (65%). Accordingly, the TOFH is a desirable feedstock applicable for bioprocessing of the renewable biomass like other common lignocellulosic biomass. The AAO–AHP pretreatment process constructed herein has presented an outstanding feature of good selectivity on the TOFH feedstock.

AAO–AHP-pretreated substrates have presented appealing structural features, i.e., big specific area and low lignin contribution, towards a good susceptibility and accessibility to cellulase enzymes. Thus, the substrate has presented a superior hydrolyzability, with enzymatic hydrolysis attaining at 85% at 3 FPU/g DM for 48 h. To our knowledge, there is rare information reported on such the good hydrolyzability. Accordingly, the hydrolyzability of AAO–AHP-pretreated substrates is at a relatively good level, which is attractive for the current lignocellulosic biomass refining industry. The AAO–AHP pretreatment possesses a superior advantage at modifying the structure and improving the hydrolyzability of substrates.

During the AAO pretreatment, components amounting to 70% of the original feedstock were dissolved into the pretreatment liquor. Besides the known substances (xylose, lignin, and glucose), other undetected components presumably involved xylan, glucan, lipid, tea saponin, and pectin in the AAO pretreatment liquor [6, 7]. Hereinto, several studies have showed the separation process of lignin and hemicellulose in the AAO pretreatment liquor [8]. Hence, the lignin and hemicelluloses are of big promise to separate from the pretreatment liquor for use [15, 38]. In the next work, accordingly, the separation of these dissolved components would be attached to importance for the value-added utilization.

## Conclusions

The TOFH, a main byproduct of tea oil processing industry is promisingly applicable as renewable lignocellulosic feedstock. The AAO–AHP pretreatment has presented an outstanding feature in selective fractionation of the lignocellulosic biomass, extremely effective to improve the substrate hydrolyzability. The AAO–AHP pretreatment can be a desirable candidate for the current pretreatment.

## Methods

### Materials

The TOFH was supplied by National Camellia Project Technology Research Center in Changsha, Hunan Province, China. Before the AAO pretreatment, the TOFH was milled and treated with aqueous ethanol for extraction of the tea saponin and tannin according to our previous method [7]. The AEO-extracted TOFH was air-dried (6% of moisture) and used as the original feedstock, containing 15.0% cellulose, 25.0% hemicellulose, and 24.6% lignin in this study. Commercial cellulase preparation, Cellic CTec2 (150 FPU/mL) was presented by Novozymes (China) Investment Co. Ltd.

### Experimental methods

#### AAO pretreatment

The pretreatment was conducted in a reactor comprising an air bath incubator, a centrifugal rotation with eight stands and eight vessels (Keli Automation, Yantai City, China). The vessel was manufactured from stainless steel (SUS 316) and had a capacity of 200 mL. In the experiment, the air-dried substrate of 10.5 g was mixed with acetic acid solution of 100 mL in the vessel, in which sulfuric acid was added as a catalyst [8]. The vessel was air-heated at a fixed temperature for some time under the closure condition. At the end of the holding time, the vessel was pulled out of the air bath incubator and put in an ice-water bath to cool down. Then, the reaction mixture was separated by filtration through G_3_ glass filter (100 mL, pore size 15–40 μm) and washed twice with 400 g (50%) aqueous acetic acid [9]. After wash with distilled water twice (200 g/wash), the insoluble solid fraction was divided into two parts. One part was conserved in a sealed bag at 4 °C for the further enzymatic hydrolysis, and the other was dried at 105 °C to determine the pretreatment yield, main composition, and structural feature. Experiment with each sample was performed in duplicate, with the average value reported. The standard deviation is <3%.

#### Optimization of AAO pretreatment

Given the influence of such variables as pretreatment temperature, pretreatment time, acetic acid concentration (v/v), and H_2_SO_4_ addition (w/v), the AAO pretreatment was optimized with a series of experiments of a Plackett–Burman design (PBD), a steepest ascent design and a central composite design [39, 40]. The most prominent parameters and the optimal concentrations domain were tested using a Plackett–Burman design (Additional file 1: Table S1) and a steepest ascent experiment, respectively. For the central composite design experiments of three factors, there were 14 experiments augmented and six replications at the center values (zero level) to evaluate the pure error, as shown in Additional file 1: Tables S3 and S4.

Using the software Design Expert-8.0, the response surface model was obtained and confirmed for a second-order model by statistical analysis. Statistical analysis of the model was performed to evaluate the analysis of variance (ANOVA). The quality of the polynomial model equation was judged statistically using the coefficient of determination *R* ^2^ and adjustment *R* ^2^, and its statistical significance was determined by *F* value and *P* value. The significance of the regression coefficients was tested by some parameters, such as coefficient of variation (CV) and adequate precision.

#### AHP pretreatment

Undissolved solid fraction after the AAO pretreatment, namely AAO-pretreated substrate was followed by AHP pretreatment. In the study, the H_2_O_2_ solution was adjusted to pH 11.5 using NaOH [13, 17]. The AHP pretreatment was conducted with 12.5% of the solid content in an incubator for 12 h at room temperature. Under controlled condition, an optimal AHP concentration was selected at 0–5% (v/v) [14, 16]. After the AHP pretreatment, the solid and liquid fractions were separated by filtration as the above AAO pretreatment. The solid fractions were washed until neutral with the hot water (60 °C), and then dried at 60 °C overnight for subsequent study. All experiments were performed in duplicate under the same condition and average values are reported. The standard deviation is <3%.

#### Enzymatic hydrolysis

Each individual sample, approximately 0.5 g dry weight, of AEO, AAO, and AAO–AHP-pretreated substrates, was put into a 100 mL flask and suspended quickly with 25 mL (10 mL) citric buffer (0.05 M, pH 4.8) to acquire the slurry with 2% (5%) solids content (w/v) [33, 41]. The slurry was then supplemented with the cellulase preparation Cellic CTec2 at some enzyme loadings (FPU/g dry mass). The enzymatic hydrolysis was performed in an incubator (50 °C, 150 rpm) for 48 h. And the enzymatic hydrolysis (%) would be used to evaluate the hydrolyzability of various materials. Experiment with each sample was performed in duplicate, with the average value reported. The standard deviation is <3%.

#### Characterization on the structural feature of substrates

Before the analysis, all the wet samples from AAO and AAO–AHP pretreatment were parted manually into small fragments and dried to a constant weight at 60 °C. Changes in morphology of the samples were observed with a scanning electronic microscopy (SEM) (Quznfa-200, FEI, Netherlands) operated at 10 kV acceleration voltages. Morphological change of the sample was further observed with a confocal laser scanning microscopy (CLSM) (LSM 710, Zeiss, Germany) [7]. Sections of the tissue were observed directly by autofluorescence without staining. Thirty-two scans were taken for each sample with a resolution of 2 cm^−1^ in the transmission mode. X-ray diffraction (XRD) pattern of the sample was carried out on a D8 (AXS, Germany) X-ray diffractometer equipped with Ni-filtered Cu Kα_1_ radiation (*λ* = 0.154 nm) at room temperature [33]. The crystallinity index (CrI) of samples was calculated. The average size of crystallite was evaluated according to the Scherrer equation.

### Analytical procedures

Cellulase activity was determined by filter paper activity. The total reducing sugars were determined by the standard DNS method. The hydrolyzability was evaluated as follows: enzymatic hydrolysis (%) = 100 × 0.9 × (g in reducing sugar) × (g in carbohydrates)^−1^. The carbohydrate and lignin content in the sample was determined by a two-step acid hydrolysis method (NREL) [42]. The Chromaster HITACHI HPLC system equipped with an Aminex HPX-87H column (300 mm × 7.8 mm, Bio-Rad, US) and the RI detector was used to detect glucose and xylose at 60 °C of the column temperature, with 5 mM H_2_SO_4_ as the mobile phase at a flow rate of 0.6 mL/min. The pretreatment yield (%) = 100 × (g of pretreated solid) × (g of feedstock)^−1^. The cellulose retention (%) = 100 × (g in cellulose of pretreated solid) × (g in cellulose of feedstock)^−1^. The component (hemicellulose/lignin) removal (%) = 100 − 100 × (g in component of pretreated solid) × (g in component of feedstock)^−1^. All experiments were performed in duplicate under the same condition and average values are reported. The standard deviations are <2%.

## Additional file

**Additional file 1: Table S1.** PBD experimental design of the AAO pretreatment. **Table S2.** Analysis of PBD design on optimization of each variable. **Table S3.** Experimental range and levels of independent variables. **Table S4.** Full factorial CCD matrix of three variables in coded units and the experimentally observed response. **Table S5.** ANOVA results for the quadratic equation for the delignification. **Table S6.** Model coefficient estimated by multiple linear regression for the delignification. **Table S7.** Crystalline of the lignocellulosic biomass before and after the two-stage pretreatment. **Figure S1.** Predicted values versus experimental values of CCD on the delignification. **Figure S2.** SEM images of material before and after the AAO and AAO-AHP pretreatment. A, B and C means the original, AAO pretreated and AAO-AHP pretreated substance, respectively. **Figure S3.** X-ray diffraction patterns of the substance before and after the AAO and AAO-AHP pretreatment.

## Abbreviations

TOFH: tea oil fruit hull
AEO: aqueous ethanol organosolv
AGO: atmospheric glycerol organosolv
AAO: acetic acid organosolv
AHP: alkaline hydrogen peroxide
NREL: national renewable energy laboratory
DM: dry mass
FPU: filter paper unit
PBD: Plackett–Burman design
ANOVA: analysis of variance
CCD: central composite design
CV: coefficient of variation
SEM: scanning electron microscopy
CLSM: confocal laser scanning microscopy
XRD: X-ray diffraction
CrI: crystallinity index
EFB: empty fruit bunches
SEP: steam explosion pretreatment
AQA: aqueous ammonia
FA: formic acid
G: glucose
RS: reducing sugars
