# Supplementary material for: Bioprocessing of tea oil fruit hull with acetic acid organosolv pretreatment in combination with alkaline H2O2
Source: Biotechnol Biofuels. 2017 Apr 8;10:86. doi: 10.1186/s13068-017-0777-1 (PMC5385081; doi:10.1186/s13068-017-0777-1)
Supplement: Supplementary file 1 — Additional file 1: Table S1. PBD experimental design of the AAO pretreatment. Table S2. Analysis of PBD design on optimization of each variable. Table S3. Experimental range and levels of independent variables. Table S4. Full factorial CCD matrix of three variables in coded units and the experimentally observed response. Table S5. ANOVA results for the quadratic equation for the delignification. Table S6. Model coefficient estimated by multiple linear regression for the delignification. Table S7. Crystalline of the lignocellulosic biomass before and after the two-stage pretreatment. Figure S1. Predicted values versus experimental values of CCD on the delignification. Figure S2. SEM images of material before and after the AAO and AAO-AHP pretreatment. A, B and C means the original, AAO pretreated and AAO-AHP pretreated substance, respectively. Figure S3. X-ray diffraction patterns of the substance before and after the AAO and AAO-AHP pretreatment. [file 13068_2017_777_MOESM1_ESM.docx]

**ESI to Biotechnology for Biofuels**

Bioprocessing of tea oil fruit hull with acetic acid organosolv pretreatment in combination with alkaline H_2_O_2_

Song Tang ^1,2^, Rukuan Liu ^3^, Fubao Fuelbiol Sun ^1*^, Chunying Dong ^3^, Rui Wang ^3^, Zhongyuan Gao ^1,4^, Zhanying Zhang ^5^, Zhihong Xiao ^3^, Changzhu Li ^3^ and Hui Li ^3*^

^1^ Key Laboratory of Carbohydrate Chemistry and Biotechnology, Ministry of Education, School of Biotechnology, Jiangnan University, Wuxi 214122, China.

^2^ State Key Laboratory of Pulp and Paper Engineering, South China University of Technology, Guangzhou 510640, China.

^3^ National Engineering Research Center for Oil-tea Camellia, Hunan Academy of Forestry, Changsha 410004, China.

^4^ Key Laboratory of Advanced Textile Materials and Manufacturing Technology, Ministry of Education, Zhejiang Sci-Tech University, Hangzhou 310018, China.

^5^ Centre for Tropical Crops and Biocommodities, Queensland University of Technology, Brisbane, QLD 4001, Australia.

***Corresponding Author**

Fubao Fuelbiol Sun (FFS)

Key Laboratory of Carbohydrate Chemistry and Biotechnology, School of Biotechnology, Jiangnan University, Wuxi 214122, China; [fubaosun@jiangnan.edu.cn](mailto:fubaosun@jiangnan.edu.cn) ;

Hui Li (HL)

Hunan Academy of Forestry, Changsha 410004, China

Email: [lihuiluoyang@163.com](mailto:lihuiluoyang@163.com)

**Co-authors:**

Song Tang (ST), [tangsongfuelbiol@163.com](mailto:tangsongfuelbiol@163.com)

Rukuan Liu (RL), [liurukuan@gmail.com](mailto:liurukuan@gmail.com)

Chunying Dong (CD), [1219921705@qq.com](mailto:1219921705@qq.com)

Rui Wang (RW), [gjyoucha@163.co](mailto:gjyoucha@163.com)m

Zhongyuan Gao (ZG), [490509257@qq.com](mailto:490509257@qq.com)

Zhanying Zhang (ZZ), [jan.zhang@qut.edu.au](mailto:jan.zhang@qut.edu.au)

Zhihong Xiao (ZX), [xzhh1015@163.com](mailto:xzhh1015@163.com)

Changzhu Li (CL), [lichangzhu2013@aliyun.com](mailto:lichangzhu2013@aliyun.com)

**Table title and legend**

1. PBD experimental design of the AAO pretreatment.
2. Analysis of PBD design on optimization of each variables.
3. Experimental range and levels of independent variables.
4. Full factorial CCD matrix of three variables in coded units and the experimentally observed response.
5. ANOVA results for the quadratic equation for the delignification.
6. Model coefficient estimated by multiple linear regression for the delignification.
7. Crystalline of the lignocellulosic biomass before and after the two-stage pretreatment.

**Tab. S1 PBD experimental design of the AAO pretreatment**

| **Variables** | **Symbol** | **Code levels** | |
| --- | --- | --- | --- |
|  |  | Lower (-1) | Higher (+1) |
| Time (h) | X_1_ | 0.5 | 2 |
| Acetic acid concentrations (%, v/v) | X_2_ | 20 | 60 |
| H_2_SO_4_ addition (%, w/v) | X_3_ | 0.2 | 0.7 |
| Pretreatment temperature (°C) | X_4_ | 115 | 135 |

**Tab.** **S2 Analysis of PBD design on optimization of each variables**

| **Factors** | Cellulose retention | | Hemicellulose retention | | Delignification | | Pretreatment yield | |
| --- | --- | --- | --- | --- | --- | --- | --- | --- |
|  | T | P | T | P | T | P | T | P |
| X_1_ | 1.3 | 0.098 | -21.1 | 0.006 | 8.9 | 0.034 | -14.7 | 0.003 |
| X_2_ | 1.2 | 0.121 | -13.3 | 0.043 | 14.2 | 0.004 | -10.5 | 0.015 |
| X_3_ | 1.5 | 0.063 | -16.0 | 0.021 | 9.5 | 0.027 | -11.2 | 0.011 |
| X_4_ | -0.2 | 0.810 | -11.4 | 0.072 | 4.9 | 0.189 | -8.0 | 0.044 |
| **Significance of the model** | 91.7 | 0.103 | 59.2 | 0.008 | 31.0 | 0.008 | 65.1 | 0.003 |
|  | No significance | | Significance | | Significance | | Significance | |

*T*, *P* and *F* means coefficient estimate, prob and response significance, respectively

**Tab. S3 Experimental rang and levels of independent variables**

| **Variables** | **Symbol** | **Code levels** | | |
| --- | --- | --- | --- | --- |
|  |  | -1 | 0 | 1 |
| Time (h) | X_1_ | 1.4 | 1.6 | 1.8 |
| Acetic acid concentrations (%, v/v) | X_2_ | 45 | 50 | 55 |
| H_2_SO_4_ addition (%, w/v) | X_3_ | 0.51 | 0.58 | 0.64 |

**Tab.** **S4 Full factorial CCD matrix of three variables in coded units and the experimentally observed response**

| **Run** | **X_1_** | **X_2_** | **X_3_** | **Delignification (%)** |
| --- | --- | --- | --- | --- |
| 1 | 1.9 | 50.0 | 0.58 | 64.5 |
| 2 | 1.6 | 50.0 | 0.58 | 64.0 |
| 3 | 1.6 | 41.6 | 0.58 | 50.4 |
| 4 | 1.8 | 55.0 | 0.51 | 64.8 |
| 5 | 1.8 | 55.0 | 0.64 | 68.7 |
| 6 | 1.6 | 50.0 | 0.58 | 65.4 |
| 7 | 1.8 | 45.0 | 0.64 | 65.0 |
| 8 | 1.4 | 55.0 | 0.51 | 50.2 |
| 9 | 1.6 | 50.0 | 0.58 | 65.5 |
| 10 | 1.6 | 50.0 | 0.58 | 66.1 |
| 11 | 1.8 | 45.0 | 0.51 | 54.0 |
| 12 | 1.6 | 50.0 | 0.58 | 63.2 |
| 13 | 1.6 | 50.0 | 0.47 | 48.7 |
| 14 | 1.4 | 45.0 | 0.64 | 58.9 |
| 15 | 1.4 | 45.0 | 0.51 | 41.6 |
| 16 | 1.6 | 50.0 | 0.68 | 69.1 |
| 17 | 1.4 | 55.0 | 0.64 | 61.7 |
| 18 | 1.6 | 50.0 | 0.58 | 65.5 |
| 19 | 1.6 | 58.4 | 0.58 | 65.3 |
| 20 | 1.3 | 50.0 | 0.58 | 48.7 |

**Tab.** **S5 ANOVA results for the quadratic equation for the delignification**

|  | **Sum of squares** | **Freedom degrees** | **Mean square** | **F-value** | **P** |
| --- | --- | --- | --- | --- | --- |
| **Model** | 1217.73 | 9 | 135.30 | 96.21 | < 0.0001 |
| **Residual(error)** | 14.06 | 10 | 1.41 | Not clear | Not clear |
| **Lack of fit** | 8.24 | 5 | 1.65 | 1.41 | 0.3566 > 0.1 |
| **Pure error** | 5.83 | 5 | 1.17 | Not clear | Not clear |
| **Total** | 1231.79 | 19 | Not clear | Not clear | Not clear |

R^2^=0.989; CV=1.97%; Adj R^2^=0.978; Pred R^2^=0.942; Adeq Precision=34.196

**Tab.** **S6 Model coefficient estimated by multiple linear regression for the delignification**

| **Term** | **Coefficient** | **F-value** | **P** |
| --- | --- | --- | --- |
| Intercept | 64.91 | No clear | No clear |
| X_1_ | 4.88 | 231.53 | < 0.0001 |
| X_2_ | 3.74 | 135.54 | < 0.0001 |
| X_3_ | 5.72 | 317.60 | < 0.0001 |
| X_1_X_2_ | 0.40 | 0.91 | 0.3631 |
| X_1_X_3_ | -1.74 | 17.27 | 0.002 |
| X_2_X_3_ | -1.62 | 14.87 | 0.0032 |
| X_1_^2^ | -2.79 | 79.84 | < 0.0001 |
| X_2_^2^ | -2.34 | 56.28 | < 0.0001 |
| X_3_^2^ | -1.98 | 40.34 | < 0.0001 |

**Tab. S7 Crystalline of the lignocellulose before and after the two-stage pretreatment**

| **Intensity** | **Crystal size (002)** | | **Crystalline (%)** |
| --- | --- | --- | --- |
|  | *2θ* (°) | Size (nm) |  |
| The original | 21.1 | 1.5 | 21.3 |
| AAO pretreated | 21.8 | 1.9 | 41.3 |
| AAO-AHP pretreated | 21.9 | 3.8 | 52.1 |

**Figure title and legend**


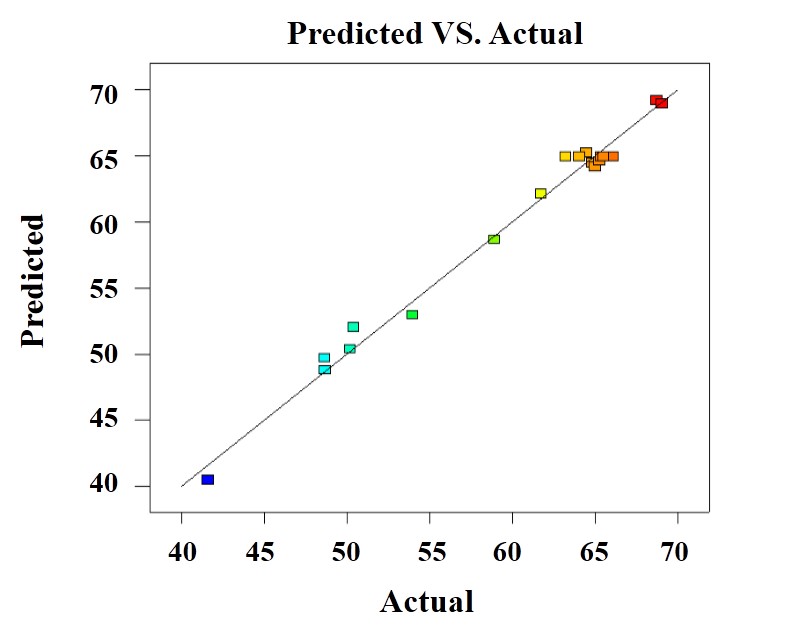


**Figure S1 Predicted values versus experimental values of CCD on the delignification (R^2^= 0.978).**


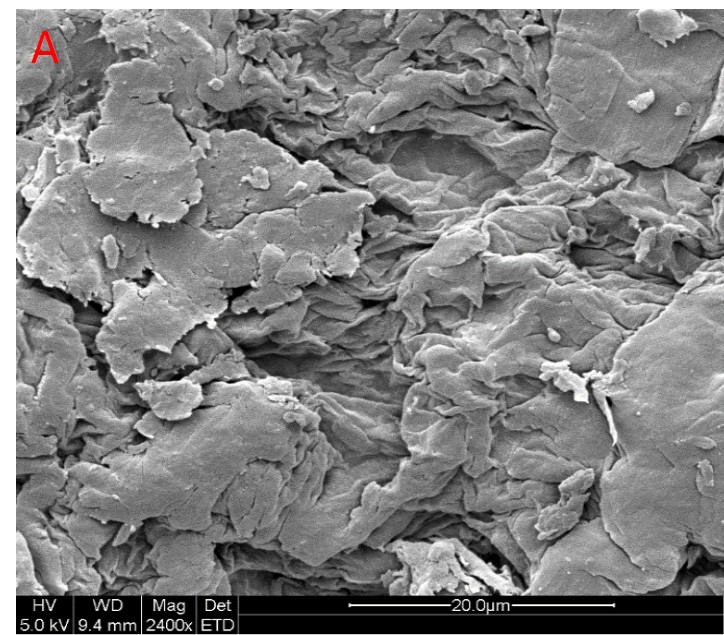

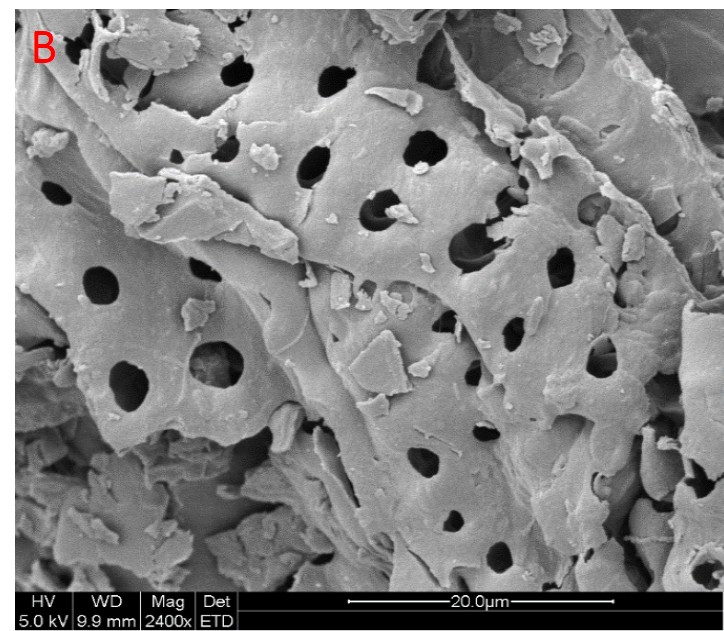

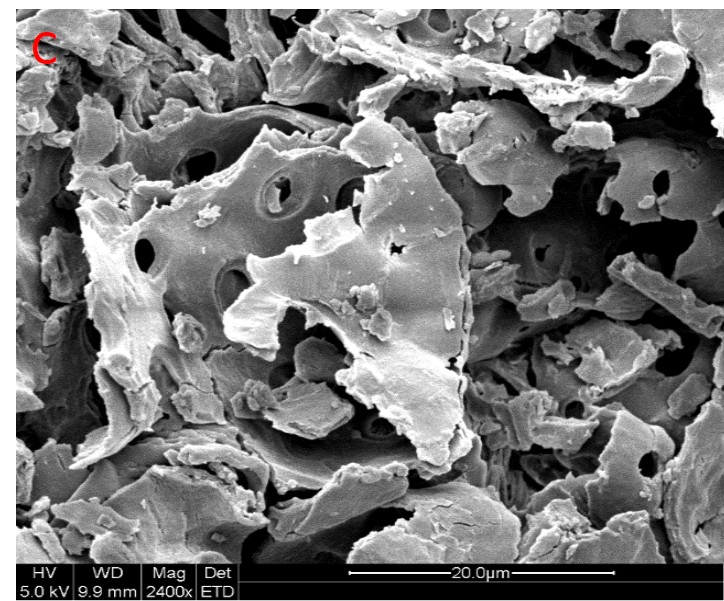


**Figure S2 SEM images of material before and after the AAO and AAO-AHP pretreatment. A, B and C means the original, AAO pretreated and AAO-AHP pretreated substance, respectively.**


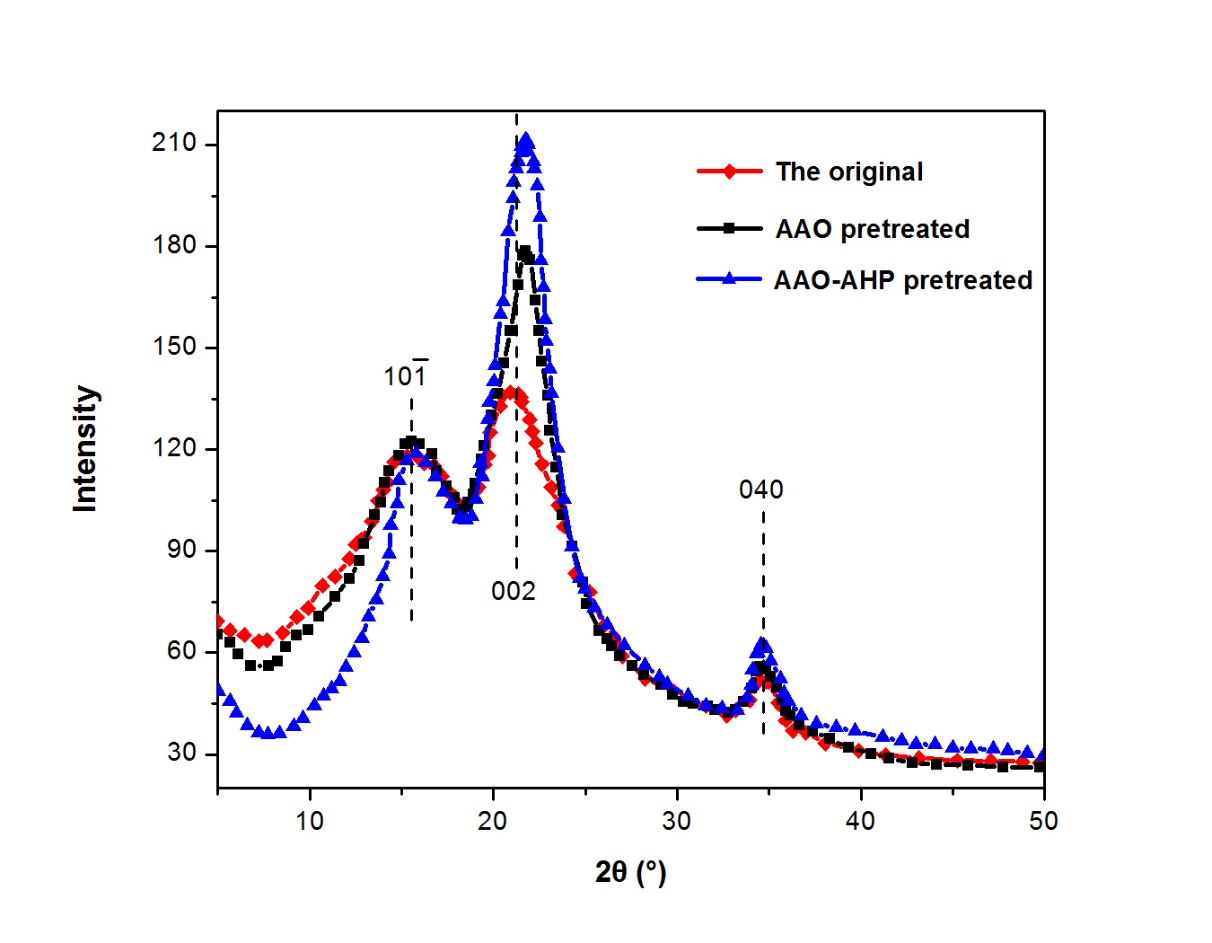


**Figure S3 X-ray diffraction patterns of the substance before and after the AAO and AAO-AHP pretreatment.**
